# Genome-wide association study and candidate gene identification for agronomic traits in 182 upward-growing fruits of *C. frutescens* and *C. annuum*

**DOI:** 10.1038/s41598-024-65332-6

**Published:** 2024-06-26

**Authors:** Genying Fu, Shuang Yu, Kun Wu, Mengxian Yang, Muhammad Ahsan Altaf, Zhuo Wu, Qin Deng, Xu Lu, Huizhen Fu, Zhiwei Wang, Shanhan Cheng

**Affiliations:** 1 Key Laboratory for Quality Regulation of Horticultural Crops of Hainan Province, School of Breeding and Multiplication (Sanya Institute of Breeding and Multiplication), Hainan University, Sanya, 572025 China; 2 Key Laboratory for Quality Regulation of Tropical Horticultural Crops of Hainan Province, School of Tropical Agriculture and Forestry, Hainan University, Haikou, 570228 China

**Keywords:** Pepper, Agronomic traits, Genome-wide association study, Single nucleotide polymorphisms, Whole genome resequencing, Sequencing, Genomic analysis, Genome-wide association studies

## Abstract

Pepper agronomic traits serve as pivotal indicators for characterizing germplasm attributes and correlations. It is important to study differential genotypic variation through phenotypic differences of target traits. Whole genome resequencing was used to sequence the whole genome among different individuals of species with known reference genomes and annotations, and based on this, differential analyses of individuals or populations were carried out to identify SNPs for agronomic traits related to pepper. This study conducted a genome-wide association study encompassing 26 key agronomic traits in 182 upward-growing fruits of *C. frutescens* and *C. annuum*. The population structure (phylogenetics, population structure, population principal component analysis, genetic relationship) and linkage disequilibrium analysis were realized to ensure the accuracy and reliability of GWAS results, and the optimal statistical model was determined. A total of 929 SNPs significantly associated with 26 agronomic traits, were identified, alongside the detection of 519 candidate genes within 100 kb region adjacent to these SNPs. Additionally, through gene annotation and expression pattern scrutiny, genes such as *GAUT1*, *COP10*, and *DDB1* correlated with fruit traits in *Capsicum frutescens* and *Capsicum annuum* were validated via qRT-PCR. In the CH20 (*Capsicum annuum*) and YB-4 (*Capsicum frutescens*) cultivars, *GAUT1* and *COP10* were cloned with cDNA lengths of 1065 bp and 561 bp, respectively, exhibiting only a small number of single nucleotide variations and nucleotide deletions. This validation provides a robust reference for molecular marker-assisted breeding of pepper agronomic traits, offering both genetic resources and theoretical foundations for future endeavors in molecular marker-assisted breeding for pepper.

## Introduction

Pepper (*Capsicum* spp.), belonging to the *Solanaceae* family, originated in tropical and subtropical regions of Central and South America^1^. It serves as a widely consumed fresh or seasoning vegetable, boasting a Chinese cultivation area exceeding 2.23 million hectares^2^. Peppers are characterized by their nutritional richness, containing significant amounts of vitamin C, capsaicin, and antioxidants, with diverse applications in culinary, medicinal, and military fields^3–5^. The consequential demand for consumption and the broad spectrum of applications underscore its significance. With a storied cultivation history and an abundance of germplasm resources, *Capsicum* comprises five cultivated species: *C. annuum* L., *C. frutescens* L., *C. chinense* Jacq*.*, *C. baccatum* L., and *C. pubescens* Ruiz & Pavon*.* Additionally, there are over 30 closely related wild species. These species exhibit substantial genetic variation in plant structure, fruit characteristics, flowers, leaves, and other traits. Agronomic traits, encompassing fruit size, shape, color, weight, and features associated with biotic and abiotic stresses, sensory attributes and nutritional qualities, constitute the foundational descriptors for genetic diversity of germplasm resources. Simultaneously, these traits serve as key breeding objectives in horticultural plants. Therefore, the localazation and mining of genes controlling these traits are of great significance for improving the yield and quality of horticultural products.

Traditionally, molecular markers such as restriction fragment length polymorphism (RFLP), random amplified polymorphic DNA (RAPD), simple sequence repeats (SSR), and linkage maps, have been employed to map numerous quantitative trait loci (QTL) related to pepper traits onto have chromosomes. Examples include fs3.1 and fs10.1^6^, governing fruit shape, and fw2.1 and fw6.1^7^, governing fruit weight. However, achieving accurate detection and identification of QTLs require higher density genetic maps. Yarnes et al. (2013) utilized a pepper gene chip encompassing 30,815 EST sequences to perform gene typing on a recombinant inbred line (RIL) population, successfully mapping 96 QTLs for 38 traits.Unfortunately, the analysis did not extend to identifying genes in the QTL region^8^. Han et al. (2016) constructed an ultra-high-density bin map of pepper using a sliding window approach, detecting 86 significant QTLs controlling 17 horticultural traits, including plant structure, leaf dimensions, flower size, fruit dimensions, and weight, Notably, they identified 32 major effect QTL sites governing 13 traits^7^. However, due to the influence of material homogeneity and low coverage sequencing, it was found that the number of gene loci controlling important agronomic traits in pepper is still relatively limited.

High-throughput resequencing of significant germplasm resources in both flora and fauna has emerged as a valuable tool for genome-wide genotyping and the execution of genome-wide association studies (GWAS). This approach involves the integration of targeted phenotype data to identify single nucleotide polymorphism (SNP) loci associated with specific phenotypes, facilitating the precise localization of genes linked to various traits. The advantages of this method include the rapid rapid identification of genetic variations, comprehensive analysis of population structure, and the generation of an extensive pool of candidate genes. Its successfully application extend across diverse crops, such as wheat^9^, maize^10^, soybean^11^, grapes^12^, cucumbers^13^, and tomatoes^14^ Colonna et al. (2019) sequenced 1.8% of the genome of 373 materials from 11 pepper species sourced from 51 countries. Their investigation illuminated genomic variations at the population level, further delineating subdivisions at both group and species levels and confirmed a gene *Longifolia 1-like* affecting fruit shape^15^. Based on the published reference genome information of peppers such as Zunla 1 and CM334 (*Capsicum annuum* L), Ahn et al. (2016) conducted a comprehensive resequencing of the entire genomes of *C. baccatum* (PRH1—a powdery mildew resistant line) and *C. annuum* (Saengryeg—a powdery mildew susceptible line) for GWAS analysis, successfully identifying 6,281 SNPs associated with 46 powdery mildew resistance genes^16^. Wu et al. (2019) utilized specific-locus amplified fragment sequencing (SLAF-seq) to perform GWAS on 287 agronomic traits of 36 pepper resources and accurately identified *Capana06g002967* and *Capana06g002969* as candidate genes for Rf^17^. Ro et al. (2023) identified 57 SNPs significantly associated with anthracnose resistance from 197 *C. chinense* through GBSand GWAS analysis^18^. In the study of important fruit traits. Du et al. (2019) employed the Target SNP-seq genotyping method to locate nine significantly associated loci with the fruit shape index among 271 pepper varieties, located on chromosomes 1, 2, 3, 4, 6 and 12^19^. Nimmakayala et al. (2021) identified 43,081 SNPs related to various fruit traits, with 12 SNPs associated with the number of locules and 8 SNPs linked to other fruit shape traits using GBS. GWAS analysis unveiled that SNPs in genes such as CLAVAT1, WD-40, Auxin receptor, AAA type ATPase family protein, and RNA polymerase III serve as primary markers for the number of locules, and others such as subunit of exocyst complex 8, enhancer of ABA co-receptor 1, tetratricopeptide-repeat thioredoxin-like 3 and pleiotropic drug resistance proteins are associated with fruit shape. Notably, CLAVAT1, WD-40 and the auxin receptor gene are established as known genes influencing tomato fruit shape^20^. Lee et al. (2022) used a combination of GWAS and QTL methods to study candidate gene for the main fruit traits of 351 chili pepper fruit, obtainings 187 significant SNP loci related to fruit, and identifying of 16 candidate genes related to the fruit^21^. It is evident that the materials and sequencing methods used are different, and there are significant differences in the number of number of identified SNP loci and candidate genes. More important agronomic trait genes in peppers still need to be discovered. Importantly, numerous genes influencing key agronomic traits in peppers are yet to be discovered.

There are many escaped peppers, and introduced cultivation pod peppers in Hainan, China, Preliminary identification shows that escaped peppers belong to *C. frutescens*, while pod peppers are *C. annuum* L., and the fruit vary in length and size, but all have characteristics such as upward-growing, high aroma and spicy taste. However, the genomic information, population relationships, and geographic distribution of the two types of peppers are still unclear^22,23^. This study endeavors to resequence 107 local escaped peppers from Hainan and 75 cultivated pod pepper (*C. annuum* var. Conoides) varieties. Through the analysis of the genetic diversity of these 182 materials, the research aimed to elucidate the distribution relationships of local escaped peppers in Hainan and utilizing GWAS, pinpoint candidate genes associated with pivotal agronomic traits. This work lays the groundwork for future gene exploration and molecular breeding efforts.

## Results

### Diversity assessment of major agronomic traits

**Figure 1:**
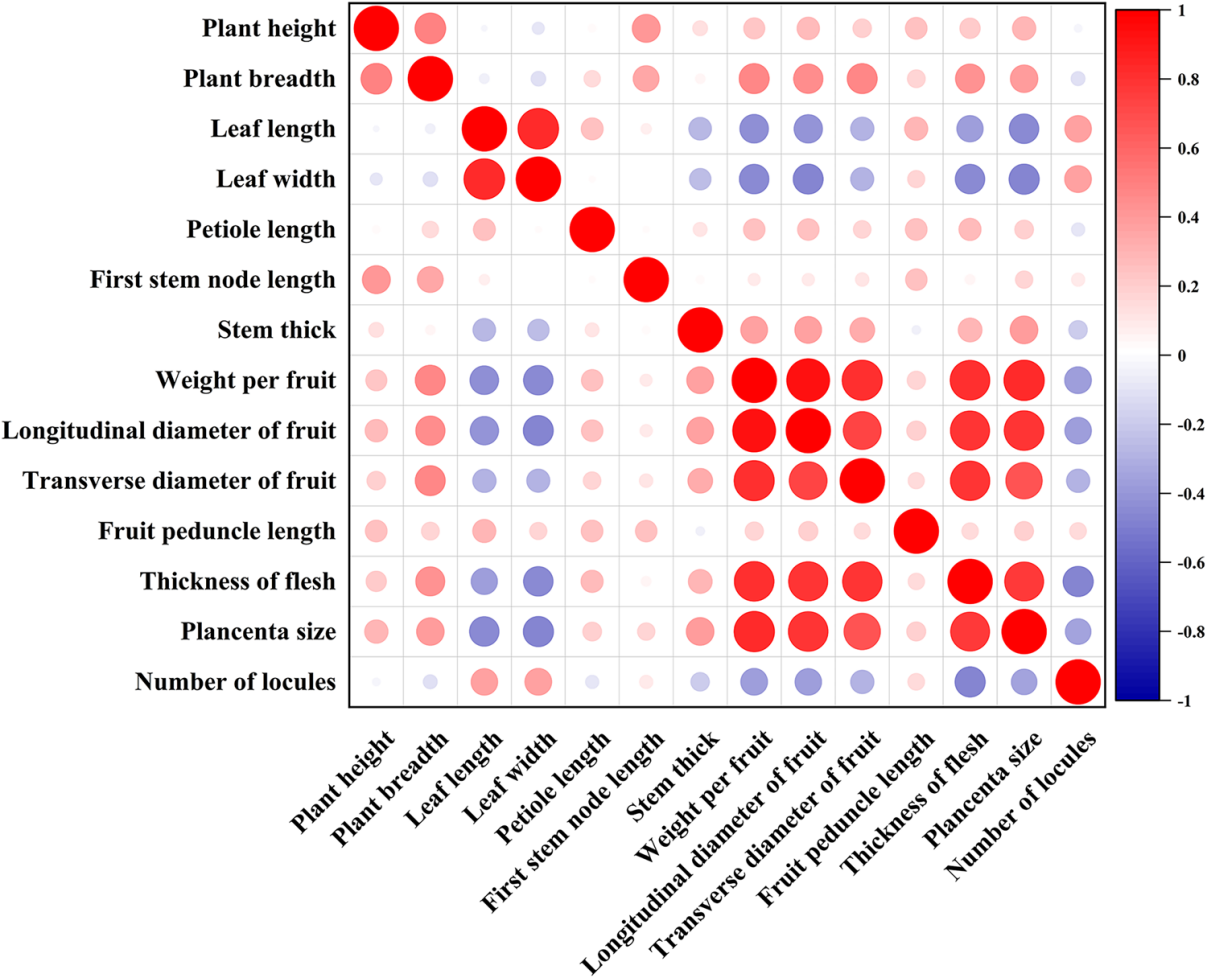
Correlation analysis of quantitative traits. Red indicates positive correlation, while blue indicates negative correlation. The intensity of color reflects strength of the correlation.

The 26 agronomic traits of peppers investigated in this study were classified into 12 quality traits and 14 quantitative traits (Tables S1–S3). Plant height (unit: cm) has a maximum value of 122.20 and a minimum value of 40.50; weight per fruit (unit: g) maximum value is 5.46, the minimum value is 0.23; longitudinal diameter of fruit (unit: mm) maximum value is 83.87, the minimum value is 15.48. The results showed that the Shannon–Wiener index for qualitative and quanlitative traits ranged from 0.95 to 2.07 and 0.03 to 0.95, (average 0.49), with mean values of 1.82 and 0.49, respectively. Except for locule number, the Shannon–Wiener index for quantitative traits surpassed that of qualitative traits, signifying a more extensive diversity in quantitative traits. The Correlation analysis of the 14 quantitative traits data indicated that there are 57 pairs of traits with extremely significant correlation between *C.frutescens* and *C. annuum* resources, of which 39 pairs are positively correlated and 18 pairs are negatively correlated. Notably, the maximum correlation coefficient between weight per fruit and longitudianl diameter of commercial fruits is 0.93 (Fig. 1). Also, the Lowest correlation coefficient of 0.001 was found between weight per fruit and plant height. (Table S4). The majority of the 26 agronomic traits demonstrated substantial phenotypic variations, affirming the suitability of the population for GWAS analysis.

### Whole genome resequencing analysis

A total of 107 escaped peppers sample and 75 pod pepper samples underwent re-sequencing, revealing a GC content ranging from 34.01 to 35.36%. The Q20 proportion fell between 96.14 and 98.6%, while Q30 exceeded 90%. Mapping rates varied from 96.85 to 99.53%, with an average of 99.02%, and an average sequencing depth of 9.62X. The average 1X coverage (minimum 1 base coverage) was 90.47%, and the average 5X coverage (minimum 5 base coverage) was 76.79% (Table S5). These results attest to the high sequencing and gene mapping quality, establishing a robust foundation for subsequent analyses.

**Figure 2:**
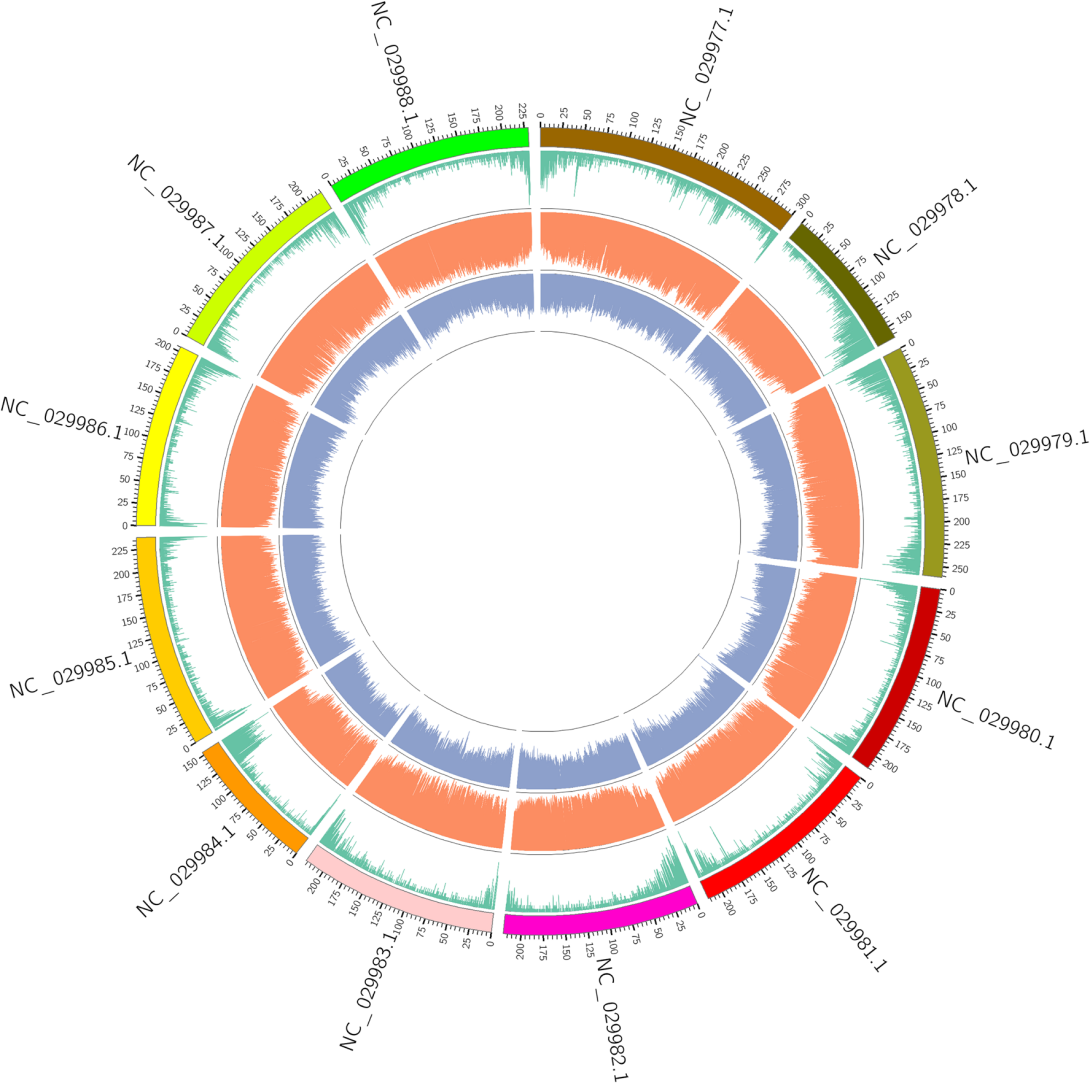
Distribution of variant types on chromosomes for each sample. Outer to inner: chromosome coordinates (colored squares), SNP density distribution (orange), and Indel density distribution (blue).

Employing the Genome Analysis Toolkit (GATK) for SNP detection yielded a total of 64,110,473 identified SNPs. The distribution of these variations was visually represented using a circos plot (Fig. 2). Uniform distribution of SNPs and Indels across chromosomes underscired indicating the reliability of the SNP and Indel data.

### Population structure and linkage disequilibrium analysis

**Figure 3:**
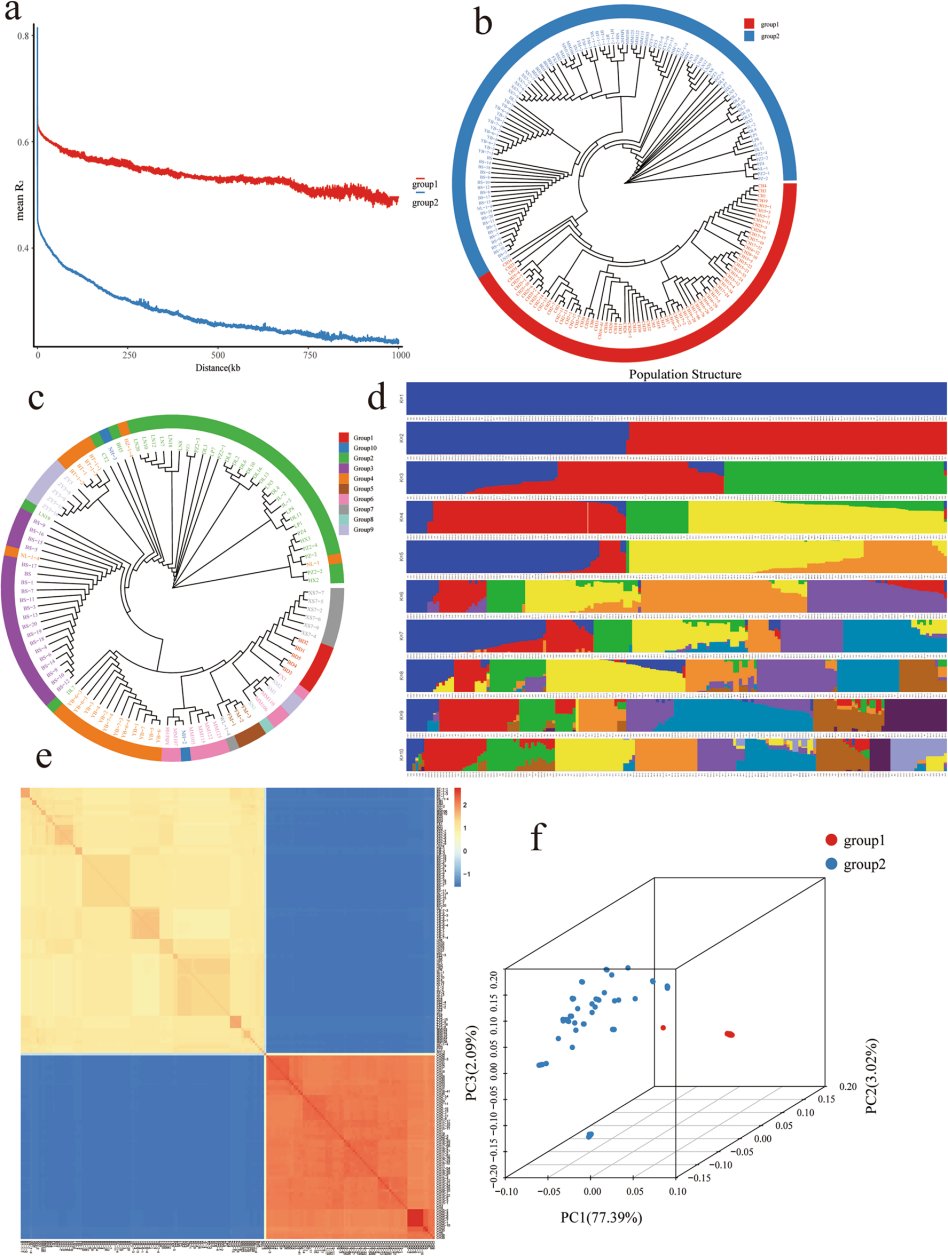
Population structure and chain imbalance analysis of *C. annuum* and *C. frutescens*. (**a**) Linkage disequilibrium analysis. (**b**) Phylogenetic analysis of 182 *C. annuum* and *C. frutescens*. (**c**) Phylogenetic analysis of 107 Hainan native escaped pepper populations (*C. frutescens*). (**d**) Population structure analysis. (**e**) Kinship analysis (**f**) Principal component analysis. Blue in A, B and F represents group 1; red represents group 2.

The population structure analysis yields insights into the subdivision of the population into distinct subgroups. Integrating population structure analysis as a covariate proves effective in mitigating false positives resulting from population structure in GWAS analysis. Assuming the population can be divided into K = 2 ~ 10 subgroups, when K = 10 (Figure S2, Table S6), the lowest cv value is observed, signifying the optimal subdivision of all pepper germplasm resources into 10 groups (Fig. 3d). Conversely, at K = 1, the population remains undivided; at K = 2, clear stratification emerges, indicating the existence of two subgroups within the 182 pepper resources under study. Subgroup 1, denoted in red, comprises 107 materials, all of *C.frutescens*,while Subgroup, represented in blue, consists of 75 materials, exclusively of *C.annuum*. This aligns with the results of the phylogenetic tree and principal component analysis.

By using the NJ method to construct a phylogenetic tree, it is possible to more intuitively present the differences in varieties and the distance of genetic relationships within a population. The phylogenetic tree shows that the 182 individuals in the pepper population in this study can be roughly divided into two branches, namely group 1 and group 2, which are consistent with the results of population structure analysis (Fig. 3b). Further systematic evolutionary analysis of 107 samples of *Capsicum frutescens* from Hainan (Fig. 3c) reveals pronounced differentiation among different cities and counties. For instance, peppers from Baisha constitute the third branch, while those from Haikou and Wenchang belong to the sixth and seventh branches, respectively. Notably, within the same city or county, peppers sourced from different towns also exhibit significant divergence, such as the second branch. Despite Dongying Town, Luni Village, and Duowen Town, Duolang Village, both being located in Lingao County, they belong to distinct branches. Principal component analysis (PCA) classifies the 182 pepper germplasms into two major groups, *C. frutescens* and *C. annuum* (Fig. 3f). PC1, PC2, and PC3 elucidate 77.39%, 3.02%, and 2.09% of the genetic variation, respectively. *C. annuum* from outside the province clusters densely, indicating a close genetic relationship among the pepper germplasms. Conversely, *C. frutescens* from diverse location within Hainan province displays greater dispersion, reflecting a rich genetic background. Evaluation of genetic relationships based on selected SNP markers (Fig. 3e) reveals genetic relationship values, with materials exhibiting of -0.2 accounting for 48.45%, those between 0 and 1.2 constituting 29.86%, and those exceeding 1.2 making up 21.68%. These findings affirm the high genetic diversity of the germplasm resources used, conducive to a robust whole-genome association study with minimal interference.

Calculation of the linkage disequilibrium (LD) decay of the 182 pepper materials (Fig. 3a) demonstrates a decrease in R^2^ with increasing physical distance. At an R^2^ of 0.1, the LD decay distance for group 1 exceeds 1000 kb, while for group 2, it surpasses 500 kb. Notably, group 1 aligns with *C. frutescens*, and group 2 with *C. annuum*, indicating a faster LD decay rate for *C. annuum*, with the smallest LD decay distance observed in the *C.frutescens* population. This further substantiates the high genetic diversity within the *C. frutescens* population.

Genome-wide association study of 26 agronomic traits.

**Figure 4:**
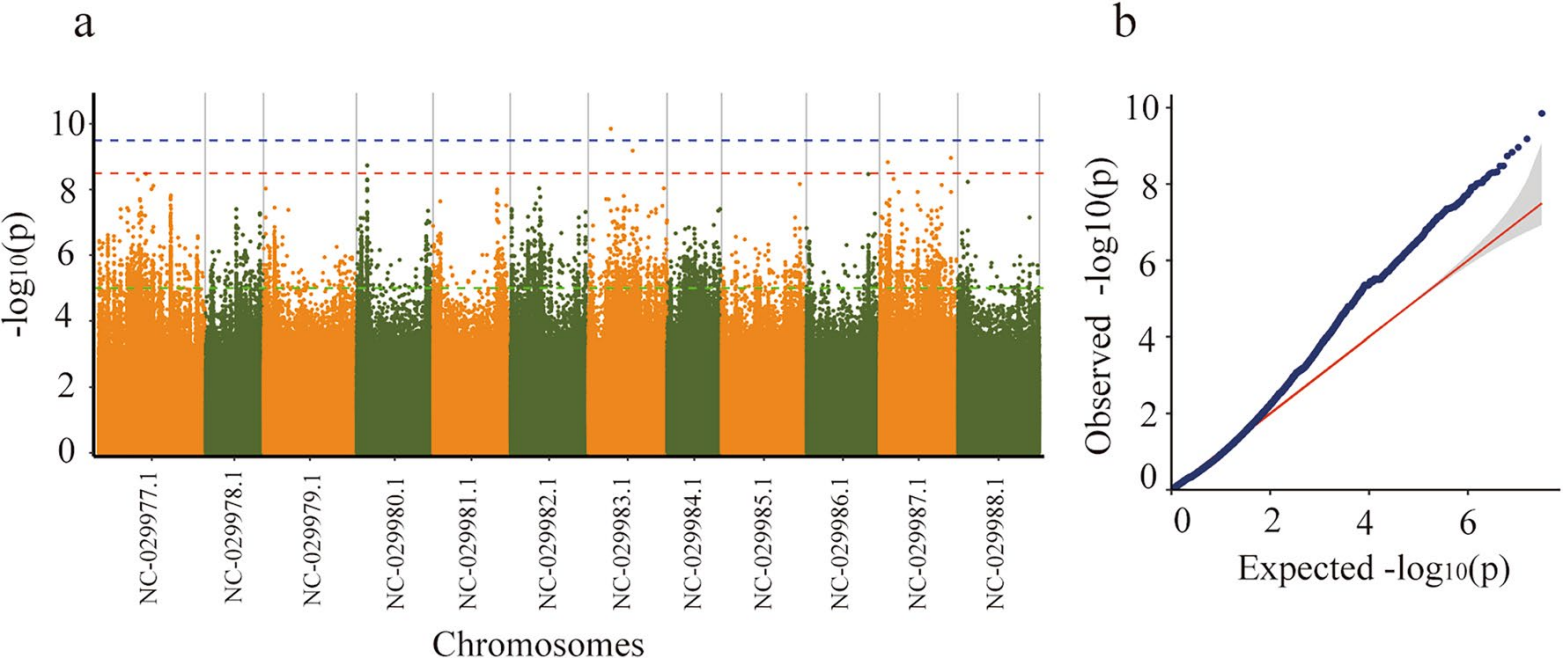
Manhattan and Q-Q plots of the LMM association model for fruit peduncle length. Horizontal coordinates represent chromosome positions, vertical coordinates represent − log10(*p*) values, with green line representing a value of 5.Blue or red denotes 0.1/labeled amount and 0.01/labeled amount. Loci above the threshold line are candidate loci.

Utilizing a subset of highly consistent SNPs derived from 182 pepper, we conducted a GWAS for 26 agronomic traits (Table 1). To enhance the reliability of GWAS results and mitigate the influence of population structure, we employed two statistical models: the Linear Mixed Model (LMM) and the Efficient Mixed Model Association eXpedited (EMMAX). After comparing their performance through Q-Q plots, we identified the most suitable statistical model for subsequent GWAS analysis of each trait. The LMM proved optimal for 9 traits, while EMMAX was optimal for 17 traits, .For instance, stem length proved suitable for GWAS analysis using the LMM model (Fig. 4, Fig. S3). The optimal model may vary for different traits, underscoring the necessity of selecting the most appropriate model for GWAS analysis for each trait. GWAS analysis was conducted on 26 agronomic traits, with results detailed in Table S8. Fifteen traits, such as Plant height, Weight per fruit, and Longitudinal diameter of fruit, exhibited 929 significantly associated loci. Conversely, Branching type, Leaf surface, Leaf shape, and eleven other traits showed no significant associations (Table S7).

**Table 1:** Screening loci and gene counts for pepper traits identified through GWAS.

Trait	Model	Chromosome	Number of SNPs	gene	− log10(p)
Plant height	EMMAX	NC_029977.1	13	26	5.000
		NC_029978.1	3		
		NC_029980.1	1		
		NC_029981.1	5		
		NC_029982.1	2		
		NC_029984.1	1		
		NC_029985.1	13		
		NC_029986.1	4		
		NC_029987.1	1		
		NC_029988.1	1		
		NW_015960393.1	1		
		NW_015960461.1	1		
		NW_015960498.1	2		
		NW_015961641.1	1		
Plant breadth	LMM	NC_029978.1	2	41	5.000
		NC_029979.1	24		
		NC_029980.1	2		
		NC_029982.1	11		
		NC_029985.1	6		
		NC_029986.1	2		
		NW_015961225.1	8		
Leaf length	EMMAX	NC_029987.1	10	6	5.0000
		NC_029988.1	1		
Leaf width	EMMAX	NC_029980.1	10	10	8.495
Petiole length	EMMAX	–	–	–	–
First stem node length/cm	LMM	NC_029978.1	1	20	5.000
		NC_029979.1	1		
		NC_029982.1	10		
		NC_029988.1	10		
		NW_015960959.1	1		
Stem thickness	EMMAX	NC_029977.1	3	7	5.000
		NC_029980.1	3		
		NC_029981.1	3		
		NC_029985.1	2		
		NC_029986.1	1		
Weight per fruit	LMM	NC_029977.1	5	46	9.495
		NC_029979.1	12		
		NC_029980.1	5		
		NC_029981.1	1		
		NC_029982.1	4		
		NC_029983.1	22		
		NC_029987.1	3		
		NC_029988.1	188		
		NW_015961202.1	1		
		NW_015961228.1	2		
Longitudinal diameter of fruit	LMM	NC_029977.1	7	62	9.495
		NC_029979.1	8		
		NC_029982.1	5		
		NC_029983.1	7		
		NC_029985.1	2		
		NC_029987.1	6		
		NC_029988.1	37		
		NW_015960681.1	3		
		NW_015960900.1	1		
Transverse diameter of fruit	LMM	NC_029979.1	12	28	8.495
		NC_029980.1	11		
		NC_029982.1	2		
		NC_029988.1	3		
Fruit peduncle length	LMM	NC_029977.1	5	64	5.000
		NC_029979.1	18		
		NC_029980.1	27		
		NC_029981.1	3		
		NC_029982.1	1		
		NC_029983.1	10		
		NC_029985.1	2		
		NC_029986.1	1		
		NC_029987.1	18		
		NC_029988.1	2		
		NW_015960759.1	7		
Thickness of flesh	EMMAX	–	–	–	–
Placenta size	LMM	NC_029979.1	7	50	9.495
		NC_029987.1	114		
Number of locules	EMMAX	NC_029977.1	2	31	5.000
		NC_029979.1	4		
		NC_029980.1	1		
		NC_029981.1	4		
		NC_029983.1	3		
		NC_029984.1	10		
		NC_029985.1	2		
		NC_029986.1	9		
		NC_029988.1	4		
		NW_015960458.1	1		
		NW_015961180.1	6		
		NW_015961815.1	1		
Branching type	EMMAX	–	–	–	–
Leaf surface	EMMAX	–	–	–	–
Leaf shape	EMMAX	–	–	–	–
Style length	EMMAX	NC_029977.1	3	26	5.000
		NC_029979.1	5		
		NC_029983.1	2		
		NC_029984.1	15		
		NC_029987.1	1		
		NW_015961188.1	1		
Flower color	EMMAX	–	–	–	–
Anther color	LMM	NC_029977.1	10	94	9.495
		NC_029979.1	5		
		NC_029980.1	8		
		NC_029981.1	3		
		NC_029982.1	14		
		NC_029983.1	11		
		NC_029985.1	30		
		NC_029986.1	9		
		NC_029987.1	2		
		NC_029988.1	4		
		NW_015960838.1	8		
		NW_015961196.1	2		
		NW_015961290.1	5		
		NW_015961339.1	9		
Pendage at blossom end	EMMAX	NC_029979.1	3	8	9.495
		NC_029981.1	1		
		NC_029983.1	1		
		NC_029984.1	5		
		NC_029985.1	2		
		NC_029987.1	1		
Color of immature fruit	EMMAX	–	–	–	–
Color of mature fruit	LMM	–	–	–	–
Persistent calyx at base of fruit	EMMAX	–	–	–	–
Fruit shoulder shape	EMMAX	–	–	–	–
Fruit shape	EMMAX	–	–	–	–

For instance, plant height was associated with a total of 49 SNPs (26 genes), predominantly distributes across chromosomes NC_029977.1, NC_029978.1, NC_029980.1, NC_029981.1, NC_029982.1, NC_029984.1, NC_029985.1, NC_029986.1, NC_029987.1, NC_029988.1, NW_015960393.1, NW_015960461.1, NW_015960498.1, and NW_015961641.1. Similarly, plant breadth exhibited 55 SNPs (41 genes), primarily located on chromosomes NC_029978.1, NC_029979.1, NC_029980.1, NC_029982.1, NC_029985.1, NC_029986.1, and NW_015961225.1. Stem thicknessness was associated with 12 SNPs(7 genes), mainly distributed on chromosomes NC_029977.1, NC_029980.1, NC_029981.1, NC_029985.1, and NC_029986.1. The first stem node length ,showed association with 23 SNPs (20 genes), primarily distributed on chromosomes NC_029978.1, NC_029979.1, NC_029982.1, NC_029988.1, and NW_015960959.1. Leaf length exhibited association with 11 SNPs (6 genes), primarily located on chromosomes NC_029987.1 and NC_029988.1, while leaf width displayed association with 10 SNPs (10 genes), mainly distributed on chromosome NC_029980.1. The style length was associated with 27 SNPs (26 genes), predominantly kurtosis on chromosomes NC_029977.1, NC_029979.1, NC_029983.1, NC_029984.1, NC_029987.1, and NW_015961188.1. Anther color exhibited association with 120 SNPs (94 genes), mainly distributed on chromosomes NC_029977.1, NC_029979.1, NC_029980.1, NC_029981.1, NC_029982.1, NC_029983.1, NC_029985.1, NC_029986.1, NC_029987.1, NC_029988.1, NW_015960838.1, NW_015961196.1, NW_015961290.1, and NW_015961339.1.

Moreover, SNP analysis identified a total of 243 SNPs (46 genes) associated with weight per fruit, mainly showing kurtosis on chromosomes NC_029977.1, NC_029979.1, NC_029980.1, NC_029981.1, NC_029982.1, NC_029983.1, NC_029987.1, NC_029988.1, NW_015961202.1, and NW_015961228.1. Additionally, 76 SNPs (62 genes) were associated with the longitudinal diameter of fruits, primarily distributed on chromosomes NC_029977.1, NC_029979.1, NC_029982.1, NC_029983.1, NC_029985.1, NC_029987.1, NC_029988.1, NW_015960681.1, and NW_015960900.1. Further, 28 SNPs (28 genes) were associated with the transverse diameter of fruits, predominantly distributed on chromosomes NC_029979.1, NC_029980.1, NC_029982.1, and NC_029988.1. Additionally, 94 SNPs (64 genes) were associated with fruit peduncle length, mainly distributed on chromosomes NC_029977.1, NC_029979.1, NC_029980.1, NC_029981.1, NC_029982.1, NC_029983.1, NC_029985.1, NC_029986.1, NC_029987.1, NC_029988.1, and NW_015960759.1. Furthermore, 121 SNPs (50 genes) were associated with placenta size, primarily distributed on chromosomes NC_029979.1 and NC_029987.1. Additionally, 47 SNPs (31 genes) were associated with the number of locules, mainly distributed on chromosomes NC_029977.1, NC_029979.1, NC_029980.1, NC_029981.1, NC_029983.1, NC_029984.1, NC_029985.1, NC_029986.1, NC_029988.1, NW_015960458.1, NW_015961180.1, and NW_015961815.1. Similarly, 13 SNPs (8 genes) were associated with pendage at blossom end, mainly distributed on chromosomes NC_029979.1, NC_029981.1, NC_029983.1, NC_029984.1, NC_029985.1, and NC_029987.1.

### Gene function annotation

We identified 26, 41, 6, 10, 20, 7, 46, 62, 28, 64, 50, 31, 26, 94, and 8 genes associated with plant height, plant width, leaf length, leaf width, first stem node length, stem thickness, weight per fruit, longitudinal diameter of fruits, transverse diameter of fruits, fruit peduncle length, placenta size, number of locules, style length, anther color, and pendage at blossom end, respectively (Table S8). Subsequently, gene functional annotation was conducted for these identified genes.

**Figure 5:**
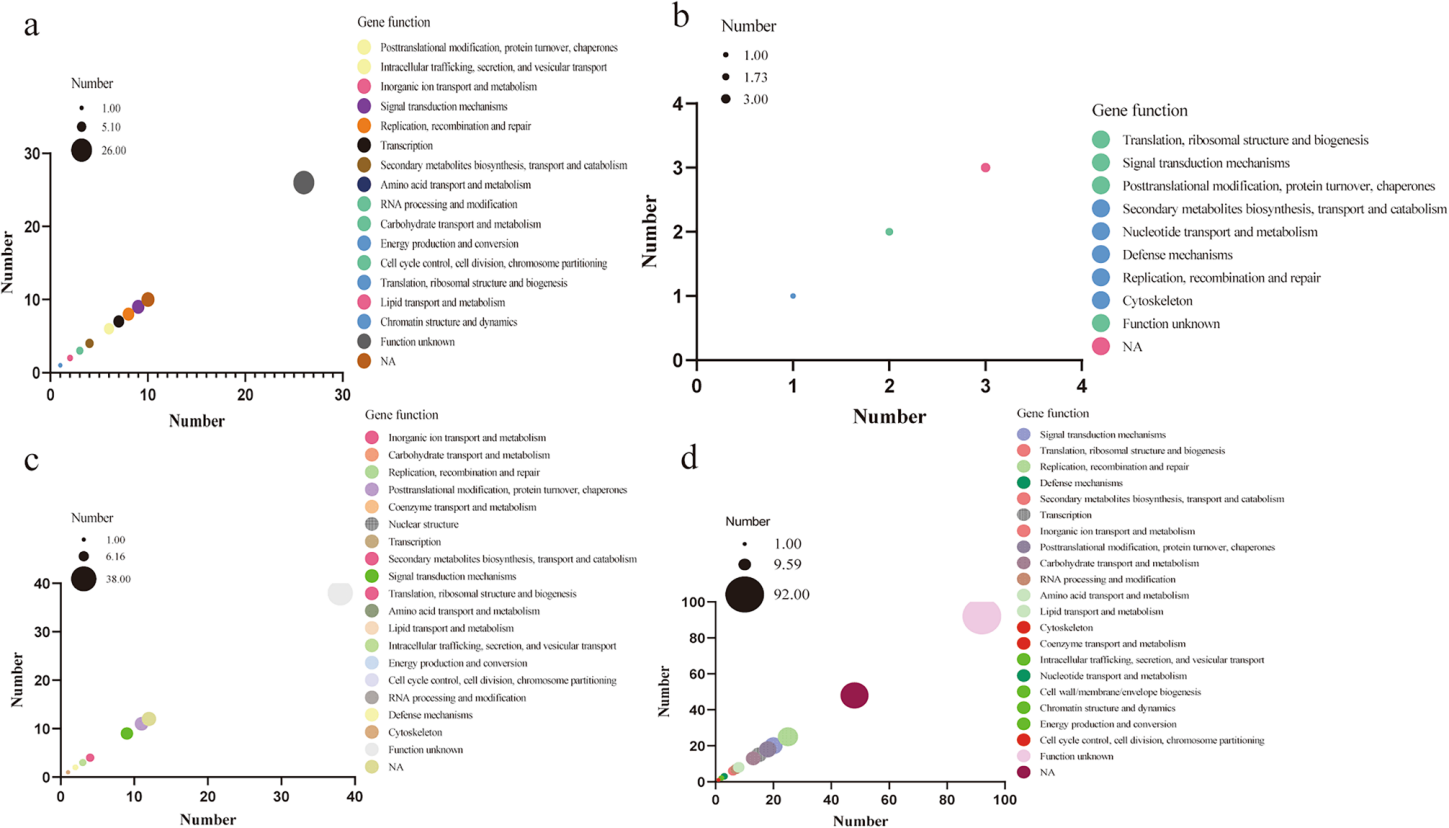
Functional annotation of 519 candidate genes. (**a**) Stem-related candidate genes. (**b**) Leaf-related candidate genes (**c**) Flower-related candidate genes. (**d**) Fruit-related candidate genes.

Genes associated with stem-related traits were primarily implicated in functions such as signal transduction mechanisms, replication, recombination and repair, transcription, posttranslational modification, protein turnover, chaperones, intracellular trafficking, secretion, and vesicular transport. Among these, 26 genes had an unknown function, and 10 genes lacked functional annotations (Fig. 5a). Regarding leaf-related traits, the annotated genes were predominantly involved in functions such as translation, ribosomal structure and biogenesis, signal transduction mechanisms, and posttranslational modification, protein turnover, chaperones, with 2 genes of unknown function and 3 genes not functionally annotated (Fig. 5b). Genes associated with flower-related traits were predominantly involved in functions such as posttranslational modification, protein turnover, chaperones, replication, recombination and repair, transcription, and signal transduction mechanisms, with 38 genes of unknown function and 12 genes lacking functional annotations (Fig. 5c). Finally, genes associated with fruit-related traits were mainly implicated in functions such as replication, recombination and repair, signal transduction mechanisms, posttranslational modification, protein turnover, chaperones, transcription, and carbohydrate transport and metabolism, with 92 genes of unknown function and 48 genes lacking functional annotations (Fig. 5d).

### Candidate gene screening and validation

**Figure 6:**
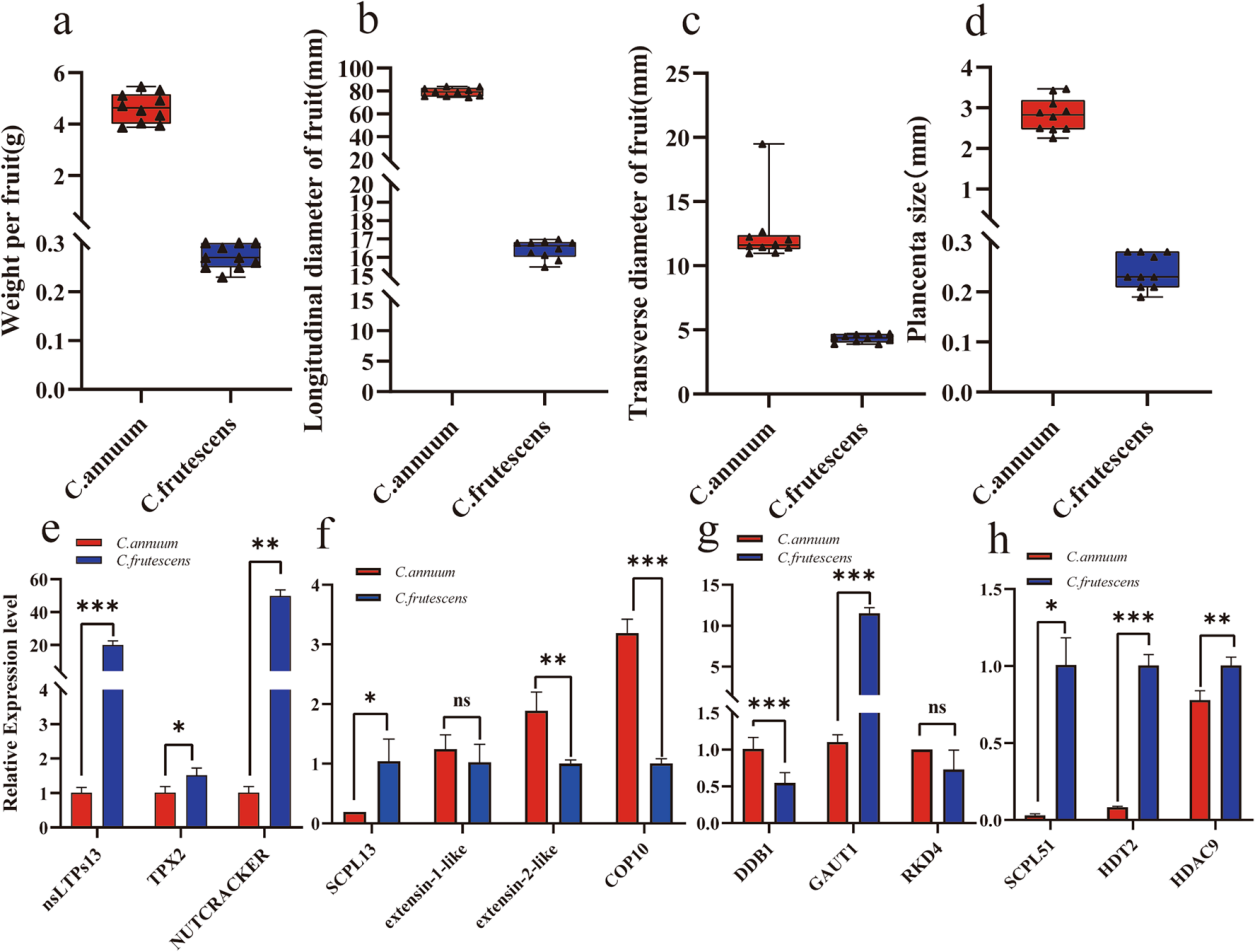
Box plots for four traits (**a**–**d**) and qRT-PCR validation results for 11 candidate genes (**e**–**h**) (**a**) Weight per fruit. (**b**) Longitudinal diameter of fruits. (c) Transverse diameter of fruits. (**d**) Placenta size. (**e**) Weight per fruit candidate genes. (**f**) longitudinal diameter of fruit candidate genes. (**g**) Transverse diameter of fruit candidate genes. (**h**) Placenta size candidate genes. * denotes significant correlation (*P* < 0.05), ** (0.001 < *P* < 0.01), and *** (*P* < 0.001) highly significant correlations.

The boxplots depicting four traits—weight per fruit, longitudinal diameter of fruits, transverse diameter of fruits, and placenta size—are illustrated in Fig. 6a–d. The numerical values of these four traits in *C. annuum* surpass those in *C. frutescens*. This, in conjunction with the qRT-PCR results from both *C. annuum* and *C. frutescens*, facilitates the analysis functional trends in genes.

Leveraging the outcome of gene functional annotation, we randomly selected 13 candidate genes associated with weight per fruit, longitudinal diameter of fruits, transverse diameter of fruits, and placenta size for qRT-PCR validation experiments shown, as depicted in Fig. 6e–h. These aimed to substantiate the influence of these genes on the growth of various traits. Notably, the expression level of the *TPX2* gene in *C. frutescens,* characterized by smaller weight per fruit, significantly exceeded that in *C. annuum,* indicating its potential role in inhibiting weight per fruit in both species. Similarly, *nsLTPs13* and *NUTCRACKER* displayed significantly higher expression levels in the variety with smaller weight per fruit, suggesting their potential as genes inhibiting weight per fruit in both *C. frutescens* and *C. annuum*. In the context of the longitudinal diameter of commercial fruits, the expression levels of *extensin-2-like* and *COP10* genes were significantly higher in *C. annuum* compared to *C. frutescens*, whereas *SCPL13* exhibited significantly higher expression in *C. frutescens.* This implies that these *extensin-2-like* and *COP10* may promote longitudinal diameter of fruits in both species, while *SCPL13* may have an inhibitory role. Notably, no significant difference in the expression level of the *extensin-1-like* gene was observed between the two varieties. Examining the transverse diameter of fruits, the expression of the *DDB1* gene in *C. annuum* with a larger diameter was significantly higher than that in *C. frutescens* with a smaller diameter, suggesting its potential role in promoting the transverse diameter of fruits in both species Conversely, the gene *GAUT1* exhibited opposite expression levels in the two varieties, indicating its potential as a gene inhibiting this trait. No significant differences in the expression levels of the *RKD4* gene were observed between the two varieties. For placenta size, *SCPL51* displayed significantly higher expression in the smaller seed cavity of *C. frutescens* compared to the larger seed cavity of *C. annuum*. In contrast, *HDT2* and *HDAC9* showed significantly higher expression in *C. frutescens*, indicating their potential roles as genes inhibiting placenta size in both varieties.

### Cloning of *GAUT1* and *COP10* and sequence analysis

**Figure 7:**
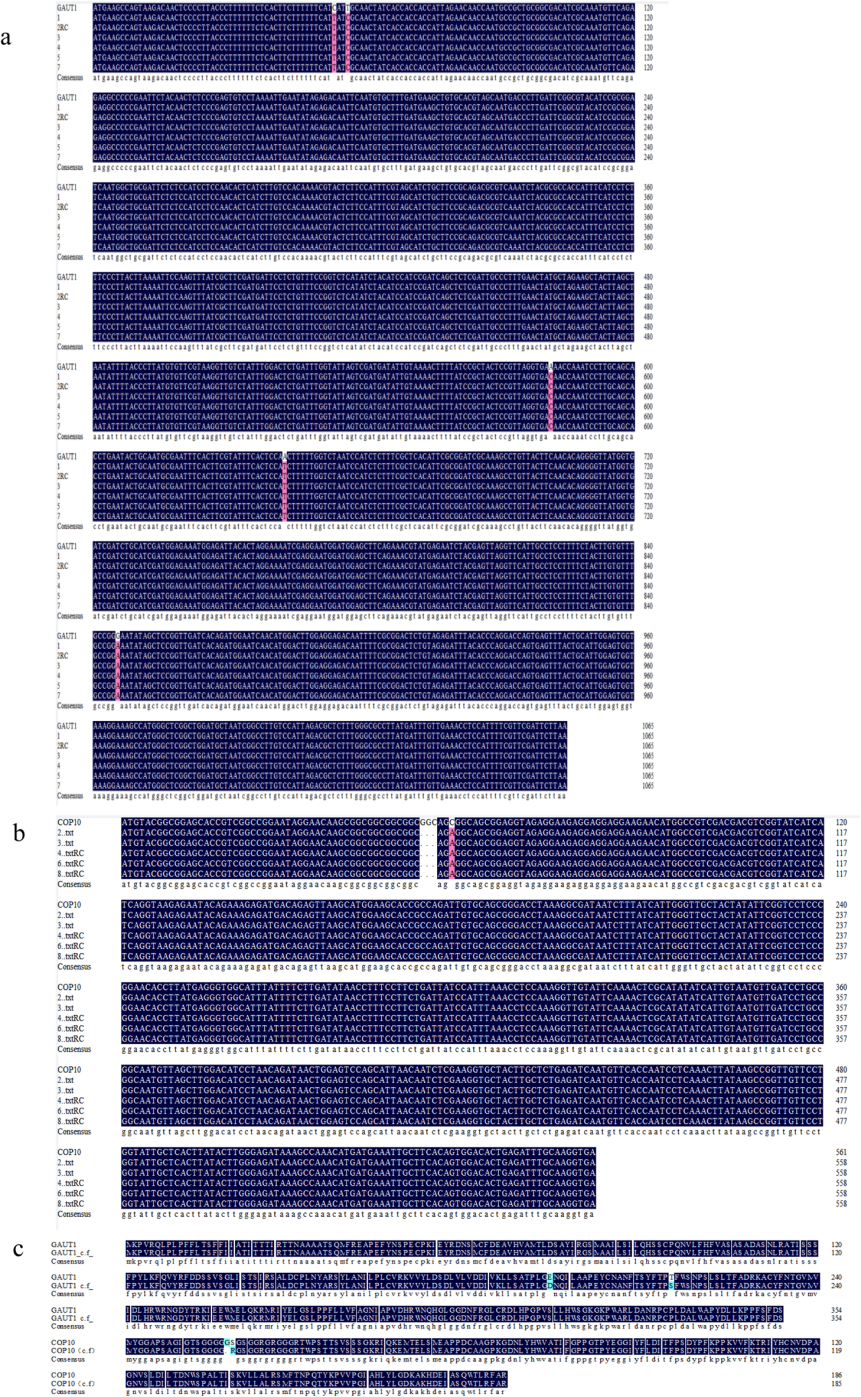
cDNA sequences and amino acid sequences of *GAUT1* and *COP10* gene clones. (**a**) Gene sequence of *GAUT1* cloned in YB-4 variety. (**b**) Gene sequence of *COP10* cloned in YB-4 variety. (**c**) Comparison of amino acid sequences of *GAUT1* gene in two clones of CH20 and two clones of YB-4. (**d**) Comparison of amino acid sequences of *GAUT1* gene in two clones of CH20 and YB-4 varieties.

In the YB-4(*C. frutescens*) variety, cloned genes *COP10* and *GAUT1* exhibit individual nucleotide mutations and deletions compared to the gene sequences cloned in CH20(*C. annuum*) (Fig. 7), which may be the reason for controlling the longitudinal and transverse diameter of chili fruits. When comparing the amino acid sequences of *GAUT1* and *COP10* between the two varieties, YB-4 has 5 single nucleotide mutation points relative to the CH20 variety. These mutations include C → T, T → C, A → C, A → T, and G → A, among which only the A → C and A → T mutations result in changes in amino acids. The A → C base mutation causes the conversion of E (glutamate) to D (aspartate), while the A → T base mutation leads to the transformation of T (threonine) into S (serine) in the *GAUT1* gene of YB-4 compared to CH20. In addition, in the *COP10* gene, YB-4 relative to CH20 exhibits 3 base deletions and 1 base mutation of C to A. The deleted bases result in the absence of a glycine in the YB-4 variety, and furthermore, the single base mutation changes S (serine) to F (phenylalanine).

## Discussion

Chili peppers exhibit notable variation in flower, leaf, and fruit-related traits, as well as diverse agronomic characteristics^24,25^. Key yield and quality attributes in chili breeding are typically influenced by multiple genes or QTL^26^, primarily used genetic mapping of agronomic traits in chili peppers has mostly focused on QTL methods^27^, the inherent low sequencing depth of this approach may lead to overlooking significant genotypes^28^. With advancements in high-throughput sequencing technology, GWAS has emerged as a potent tool for localizing candidate genes^29^. Despite the application of GWAS in chili breeding, most studies have focused on a limited number of traits or disease resistance^30–32^. In our study, GWAS was conducted on 26 agronomic traits of chili peppers, revealing significant SNPs associated with these traits. The results highlight the substantial impact of GWAS on efficiently localizing genes linked to multiple agronomic traits in chili peppers.

Agronomic traits in chili peppers encompass various aspects, including yield, quality, and resistance. Determinants of chili pepper yield include fruit longitudinal and transverse diameters, weight per fruit, and the fruit shape index. GWAS analysis is a valuable tool for localizing QTL and predicting candidate genes associated with fruit yield-related agronomic traits. This approach provides a theoretical basis for molecular marker-assisted breeding of chili peppers. In tomato research, next-generation sequencing sequencing technology has facilitated the identification of representative gene families controlling fruit size. Notably, genes such as *CNR*, *SUN,* and *OVATE*, belonging to these families, have been successfully cloned. Their roles in regulating fruit length and shape have been validated^33–36^. The insights gained from tomato studies have been applied to QTL localization in chili peppers, revealing the complex genetic structure controlling these quantitative genetic traits^37,38^. Conducting a multi-trait QTL analysis in chili pepper has identified 40 candidate genes associated with *C. annuum* traits. These genes are implicated in diverse functions, including defense response, metabolic processes oxidation–reduction, and phosphorylation^39^. Understanding the genetic basis of these traits is critical, as cell number and size are pivotal determinants of plant organ size, and any variation in these parameters can impact organ size significantly^40^. Fruit peduncle length, a crucial trait, is intricately regulated by cell number or size, influenced indirectly by hormones and multiple pathways. Kinases play essential roles in this regulation, affecting plant growth and development. In our study, SNPs related to fruit stalks were identified in key genes, including leucine-rich repeat receptor-like kinases (LRR-RLKs), serine/threonine protein kinase, ABC transporter gene, and RING finger protein. These genes are likely to play pivotal roles in growth, development, cell wall integrity, and elongation rate^41–44^. Our gene functional annotation identified genes related to plant height, first internode length, fruit length, and fruit stalk length. For instance, *nsLTPs13* plays a crucial role in cell development and organogenesis^45,46^, while *TPX2* family members are involved in various plant developmental processes^47^. *NUTCRACKER* is implicated in defining asymmetric cell division and stabilizing tissue boundaries^48^, and genes like *SCPL13*, regulate cell elongation and locule numbers in rice^49^, *Arabidopsis*^50^ and tobacco^51^.

The extensin-2-like protein actively participates in plant cell wall formation and assembly, influencing plant cell elongation functions^52^. *COP10* primarily regulates plant growth and development,with a role in light signal transduction^53,54^. The gene *DDB1,* associated wit-h commercial fruit transverse diameter, acts as a damage sensor maintaining the balance between genome integrity and cell cycle progression^8^. Galacturonosyltransferase *(GAUT)1* and *GAUT7* are core components of a plant cell wall pectin biosynthetic homogalacturonan:galacturonosyltransferase complex^55^. *SCPL51*, associated with seeds per fruit, regulates cell elongation and carpel numbers^50^. Members of the *SCP* and *SCPL* families, expressed in m-ajor tissue types, play roles in various biochemical and cellular processes in plants^56–58^. St-udies have revealed that overexpression of *SCPL41* reduces membrane lipid content, while is absence of *SCPL41* increases membrane lipid content^59^. *HDT1/2* influences the transitio-n from cell division to expansion in root tips by inhibiting the expression of GA2ox2^60^. The regulation of histone acetylation involves the antagonism between *HDAC* and histone acetyltransferases (*HAT*) , modulating enzyme activity^61^.

Our study involved the investigation of 929 significantly associated SNPs in 182 *C. frutescens* and *C. annuum*. chili pepper resources, resulting in the identification of 519 candidate genes after meticulous screening. Through qRT-PCR validation, genes such as *GAUT1*, *COP10*, and *DDB1*, associated with fruit-related traits in *C. frutescens* and *C. annuum*, were confirmed. Future endeavors will focus on identifying candidate genes and the subsequent cloning and functional analysis of genes regulating fruit length. These efforts aim to provide a solid foundation for the development of high-quality chili pepper varieties.

## Conclusions

This study conducted a comprehensive genome-wide analysis of a population comprising 182 chili pepper resources, encompassing both *C. frutescens* and *C. annuum*, through whole-genome resequencing. The analysis yieded a set of 64,110,473 high-quality SNP markers, addressing 26 key agronomic traits, including plant height, weight per fruit, fruit length, and seed cavity size. Utilizing GWAS, we identified 929 significant SNP-associated loci, unveiling 519 candidate genes. These genes play pivotal roles in plant growth and development, regulation of light signal transduction, and responses to salt stress. Furthermore, through meticulous gene annotation and assessment of expression patterns, certain candidate genes, namely *GAUT1*, *COP10*, and *DDB1*, were delineated. While these findings contribute significantly to understanding of genes related to chili pepper fruit traits, elucidating the precise mechanisms governing these traits requires further investigation. This study provides valuable genetic resources and theoretical foundation for future endeavors in molecular marker-assisted breeding of chili peppers.

## Methods and material

### Plant materials and treatment

In this study, the pepper research group at the School of Tropical Agriculture and Forestry, Hainan University. Experimental samples of hot peppers, including the collection of plant materials, were collected according to relevant institutions, national and international guidelines and legislation, with the appropriate permission of Hainan Provincial Department of Science and Technology. Systematically acquired a total of 182 samples comprising both *Capsicum annuum* and *Capsicum frutescens* (Table S9). Specifically, 107 instances of *Capsicum frutescens* were procured from diverse cities and counties in Hainan Province, It is a pre-collection of chili peppers distributed in villages, forests and ravines all over Hainan, while the remaining 75 specimens of pod pepper originated from cultivated resources in other provinces. Notably, all specimens exhibited distinctive features of tall plants and upward growth of fruits. To ensure methodological consistency,all pepper plants were cultivated in a randomized design with three replicates at Sanjia Town, Dongfang City, Hainan Province, during September 2022. Individual plant samples were tagged and tender leaves were wrapped in aluminum foil, frozen in liquid nitrogen, and stored for further use.

### DNA extraction and sequencing

The genomic DNA from tender leaves was extracted using the cetyltrimethylam-monium bromide (CTAB) method. Subsequent to extraction, the concentration and quality of the genomic DNA were determined using a NanoDrop 2000 spectrophotometer (Thermo Fisher Scientific). Following the DNA quality assessments, libraries were constructed. These qualified libraries then underwent sequencing on the NovaSeq 6000 platform. The raw reads obtained from the sequencing process underwent a series of essential data filtering steps: (1) the removal of reads containing adapters; (2) filtering out reads with more than 10% N content; (3) elimination of reads with low-quality bases exceeding 50% at a quality value below 20. This filtering process yielded clean reads, which were utilized for subsequent data analysis.

### SNP and indel calls

The Zunla-1 genome (https://www.ncbi.nlm.nih.gov/assembly/GCF_000710875.1), characterized by a size of approximately 2.9 Gb and a GC content of 34.5%, was used as the reference genome. Alignment of Clean Reads to the reference genome employed the Burrows-Wheeler Alignment (bwa-mem2 v2.2)^62^ with the MEM algorithm. The identification and removal of redundant reads were executed based on the alignment results, employing samtools (v1.9)^63^. Subsequent to alignment, the calling of SNPs and Indels was performed using the HaplotypeCaller module of GATK (v3.8). Filtering criteria encompassed parameters: QUAL < 30, QD < 2.0, MQ < 40, FS > 60.0, MQrankSum < -12.5, ReadPosRankSum < -8.0, -clusterSize 2, -clusterWindowSize 5. Further filtering of the identified SNPs was conducted based on minor allele frequency (MAF: 0.05) and site integrity (INT: 0.8), ensuring the acquisition of highly consistent SNP positions.

### Population evolutionary analysis

The MEGA X^64^ software was utilized to construct phylogenetic trees for 182 species using the neighbor-joining method, and applying the Kimura 2-parameter model with 1000 bootstrap repetitions. The analysis of population structure for the investigated materials utilized the admixture^65^ software based on SNP data. Clustering, spanning a predefined range of subgroups (K values: 1–10), determined the optimal number of subgroups based on the valley of the cross-validation error (CV error).To facilitate evolutionary analysis by revealing clustering patterns, principal component analysis was executed using the EIGENSOFT software, leveraging SNP data. Estimation of relateness between individuals in natural population was carried out using the GCTA^66^ software. Additionally, the calculation of linkage disequilibrium between pairwise SNPs within a specified distance range (1,000 kb) on the same chromosome was accomplished with the PopLDdecay(v3.41)^67^ software.

### GWAS

Filtered SNPs underwent GWAS. Wherein all traits underwent comprehensive analysis utilizing GEMMA (LMM model) and EMMAX (EMMAX model) software, incorporating both phenotype and genotype data. The formulations for these models are as follows:

1 $$ {\text{LMM}}\,{\text{model}}\,{\text{formula:}}\, Y = W\alpha + X\beta + X\mu + {\text{e }}$$

2 $$  {\text{EMMAX}}\,{\text{ model}}\,{\text{ formula: }} \begin{gathered} y_{i} = \beta_{0} + \beta_{k} X_{ik} + \eta_{{{\text{i}}\overline{k} }} \hfill \\ \hfill \\ \end{gathered}$$

### LMM model formula

#### EMMAX model formula

In these formulas, the calculation of sample-relatedness μ was performed as a random effect using GEMMA software. In cases where covariates the covariates, are present, denoted as W, they are treated as fixed effects. Here, X represents genotype, and Y represents phenotype. The parameters include β_0_ as the fixed effect, β_k_ as the marker effect, and η signifying the error term. Ultimately, each variant locus yielded an association result. The P-values derived from the whole-genome association analysis were subsequently employed to generate Manhattan and QQ plots. The selection selection of candidate thresholds was predicated on values of 0.1 and 0.01 divided by the number of valid marker loci post-quality control measures. Exploring additional candidate regions involved considering values below − log10 (*p*) = 5 as potential regions of interest.

#### Validation of candidate genes using real-time quantitative PCR (qRT-PCR)

Statistical analysis of data,encompassing box plots and qRT-PCR, was conducted aceoss a spectrum of 20 varieties. This selection comprised both the top and bottom 10 with respect to the numerical values associated with each trait. Total RNA underwent reverse transcription to cDNA following the protocols outlined in the Roche reverse transcription reagent kit. Primer sequences corresponding to genes such as *SCPL13*, *COP10*, and *DDB1* (Table 2) were designed using Premier 5.0 software, with Actin (Accession number: AY486137.1) serving as the reference gene. Subsequent qRT-PCR reactions utilized SYBR® Premix Ex Taq II, and the ensuring data analysis adhered to the 2^–ΔΔCT^ method.

**Table 2:** qRT-PCR primer information.

Gene ID	Name	Forward (5′ to 3′)	Reverse (5′ to 3′)
gene-LOC107878351	nsLTPs13	TGTAGATGGAACCGTTGAGAA	TACGAGACTGGCGATAATGAG
gene-LOC107878353	TPX2	TGAGGAGGAAATGATGGCTA	GGAGGTCGTGCTCTAAGTGA
gene-LOC107878392	NUTCRACKER	AATATGGGTGTGGGTCAAGAG	GATGGAATAAGGGTAGTCGTGT
gene-LOC107851634	SCPL13	CTTCCTTTTTATCTTGAGACCG	AGCCAATGGACCTACTTCGT
gene-LOC107854462	extensin-1-like	TTGTAGCCAGCCATGTTGTT	TTGAAGGAGCGGGTGATTT
gene-LOC107873714	extensin-2-like	AGCTTTGGAAATTTAGGGCA	GGGAGCATCATAGTTGGACG
gene-LOC107873729	COP10	CGTCGGTATCATCATCAGGT	CAAGAAAATAAATGCCACCC
gene-LOC107867065	DDB1	AAGTATCTCCGCTGCTATGTG	TAGTAGGTGAAGACCCCCGT
gene-LOC107867061	GAUT1	TCCATCTCTTTCGCTCACAT	CCATTCCTCGATTTTCCTAGT
gene-LOC107865044	RKD4	CAATCACTTGCCGATTTTTG	GCTCTTCTTATGCCTCCCAG
gene-LOC107847544	SCPL51	GGTTGGAAAACTAAAGTGGGA	GCTGGTGATTGTGTGATGCTA
gene-LOC107848037	HDT2	CCTAAAAGGGTTGAGGAGAAG	GTTGTTACCATTGGCTGCTG
gene-LOC107847639	HDAC9	ATGGAAAATCTTCGTTGCCT	TCGCCATCATAATACTCGTCA
LOC107873556	Actin	GCTGGAGGTGTATTTTTGGTT	TTGGCCCTGTCAGTCTTGTA

#### Cloning of GAUT1 and COP10

Gene full-length cloning primers were designed using Primer5.0 software based on the gene ID obtained from genomic data. The primers used were as follows: GAUT1-F, 5′-ATGAAGCCAGTAAGACAACTCCCC-3′ and GAUT1-R, 5′-TTAAGAATCGAACGAAAATGGAGG-3′ for *GAUT1* gene; COP10-F, 5′-ATGTACGGCGGAGCACCG -3′ and COP10-R, 5’-TCACCTTGCAAATCTCAGTGTCC-3′ for *COP10* gene. The full-length sequences were amplified by PCR using 2 × Phanta^@^Max Master Mix enzyme, and the correct bands were excised and purified using the Norvazo purification kit (DC301). The purified PCR products were then ligated with a TA cloning vector and transformed into competent Escherichia coli DH5α cells. Single colonies were picked for liquid PCR screening, and strains with correct and bright bands were selected for sequencing analysis by Nanshan Biotechnology Co., Ltd.

### Statistical analysis

Data analysis involved the utilization of Microsoft Excel 2007 for data organization and the calculation of the frequency distribution for qualitative traits. Additionally, the maximum, minimum, and coefficient for variation of quantitative traits were calculated. Quality traits include branching type, leaf surface, leaf shape, style length, flower colour, anther colour, appendage at blossom end, colour of immature fruit, colour of mature fruit, persistent calyx at base of fruit, fruit shoulder shape and fruit shape. Quantitative traits included plant height, plant breadth, leaf length, leaf width, petiole length, first stem node length, stem thickness, weight per fruit, longitudinal diameter of fruit, transverse diameter of fruit, fruit peduncle length, thickness of flesh, plancenta size and number of locules. Standard deviation (SD) and mean (Mean) served as classification criteria, with each 0.5 SD interval designated as a level "i." Levels were systematically categorized from i = 1 to 10, and the frequency distribution P_i_ for each level was determined. The computation of the Shannon–Weaver diversity index (H’) was undertaken using the following formula:

3 $$ H^{\prime } = - \sum\limits_{i - 1}^{s} {\left( {P_{{\text{i}}} \ln \left( {P_{i} } \right)} \right)} $$

### Supplementary Information

Supplementary Information 1.

Supplementary Information 2.

Supplementary Information 3.

Supplementary Information 4.

Supplementary Information 5.

Supplementary Information 6.

## Data Availability

The data stored in our system is accessible, but requires the author's permission. Information of relevant data can be obtained by contacting S.C. via email, 990865@hainanu.edu.cn.
