# Supplementary material for: Genome-wide association study and candidate gene identification for agronomic traits in 182 upward-growing fruits of C. frutescens and C. annuum
Source: Sci Rep. 2024 Jun 26;14:14691. doi: 10.1038/s41598-024-65332-6 (PMC11208541; doi:10.1038/s41598-024-65332-6)
Supplement: Supplementary file 1 — Supplementary Information 1. [file 41598_2024_65332_MOESM1_ESM.docx]

**Supplementary Material**

**1 Supplementary Figures and Tables**

**1.1supplementary Figures**

A field study of the important agronomic traits of chili peppers was conducted, with reference to the "*Guidelines for Descriptor Development and Data Standardization for Capsicum Germplasm*."


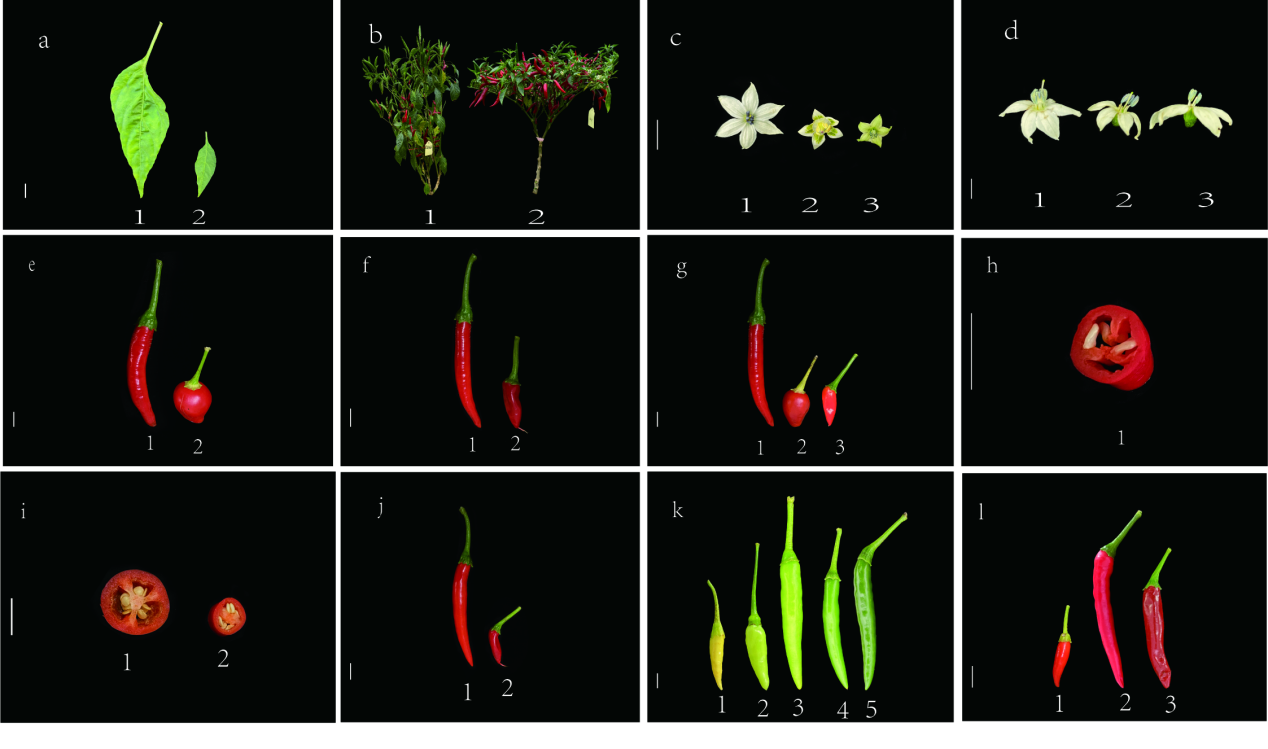


Supplementary Figure 1:A total of 182 materials were surveyed for agronomic traits related to chili peppers, including:(a) Leaf surface (1. Smooth, 2. Slightly wrinkling), leaf shape (1. Ovate, 2. Lanceolate), and leaf size.(b) Branching type (1. Limited bIndeterminate branching, 2. Determinate branching).(c) Flower color (1. White, 2. White with yellow, 3. Light green) and anther color (1. Light purple, 2. Yellow, 3. Green).(d) Style length (1. Longer than stamen, 2. Nearly the same level as stamen, 3. Shorter than stamen).(e) Persistent calyx at base of fruit (1. Sreading, 2. Covering down) and Fruit shoulder shape (1. Pointed, 2. Blunt).(f) Fruit peduncle length, longitudinal diameter, and transverse diameter of fruit.(g) Fruit shape (1. Long finger-shaped, 2. Spherical, 3. Short finger-shaped).(h) Thickness of flesh and placenta size.(i) Number of locules.(j). pendage at blossom end (1. Absent, 2. Present).(k) Color of mature fruit (1. Milk yellow, 2. Light green, 3. Green, 4. Dark green, 5. Yellowish green).(l) Color of mature fruit (1. Tangerine, 2. Bright red, 3. Dark red).The white bar represents 1 cm.


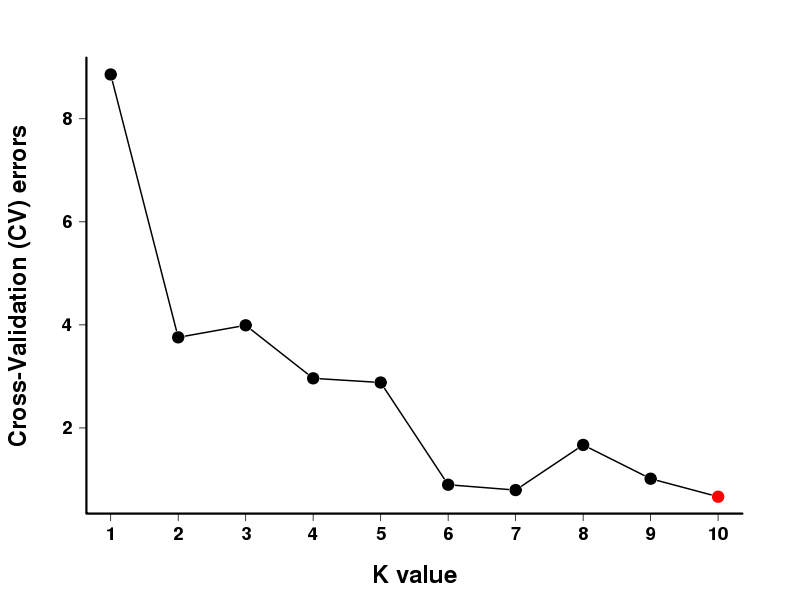


Supplementary Figure 2 Admixture Cross-validation error rate for each K-value
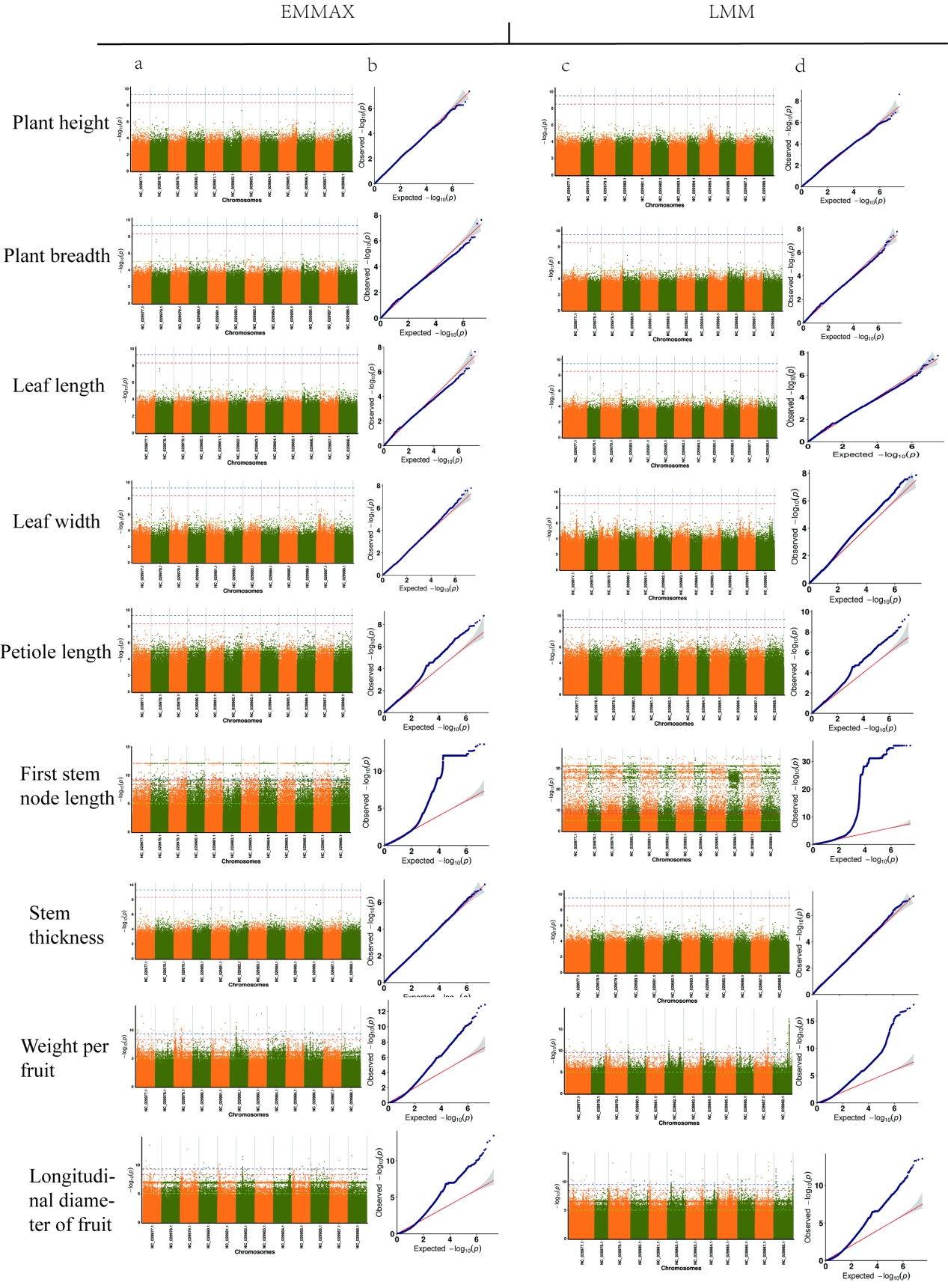


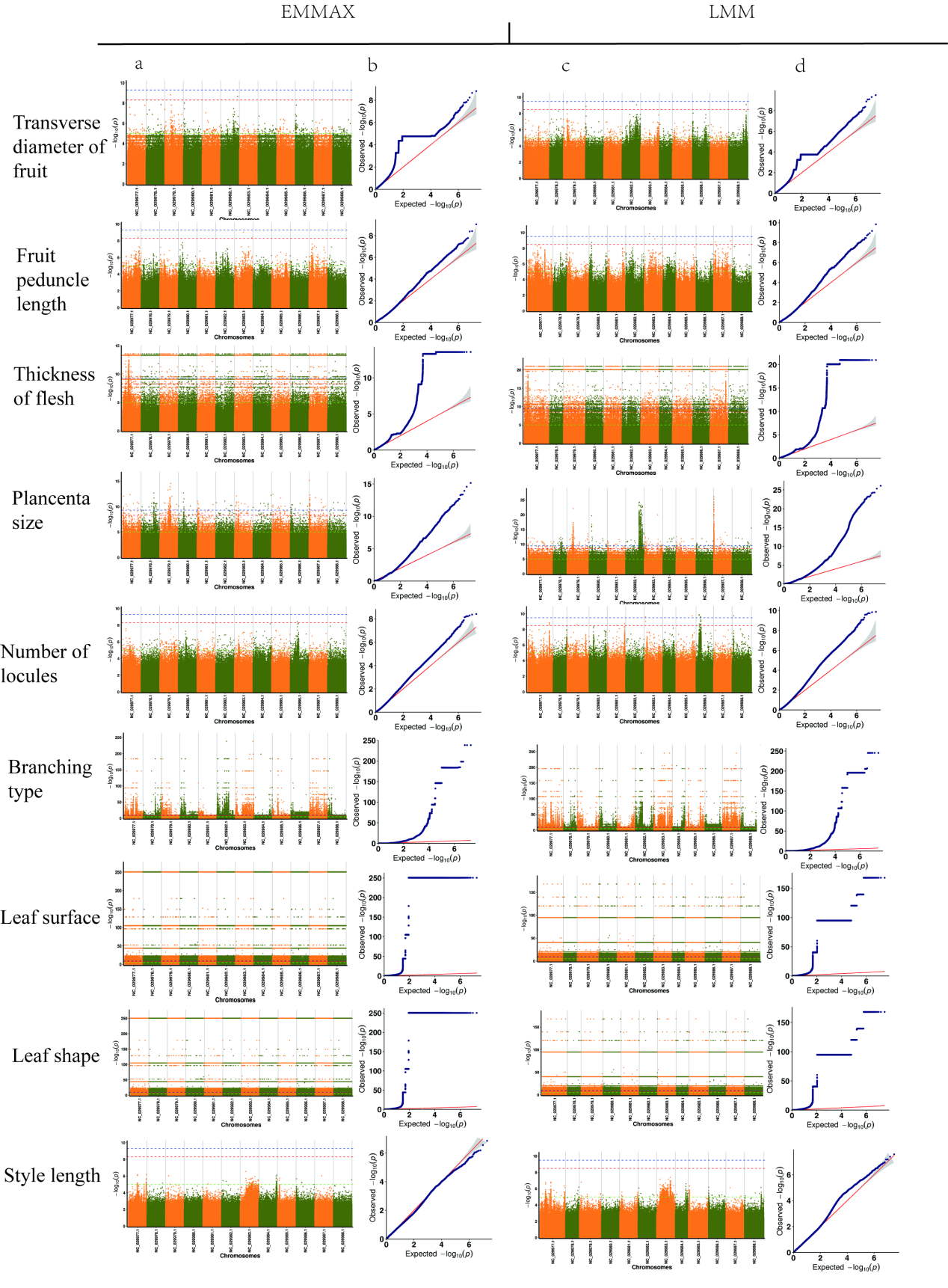


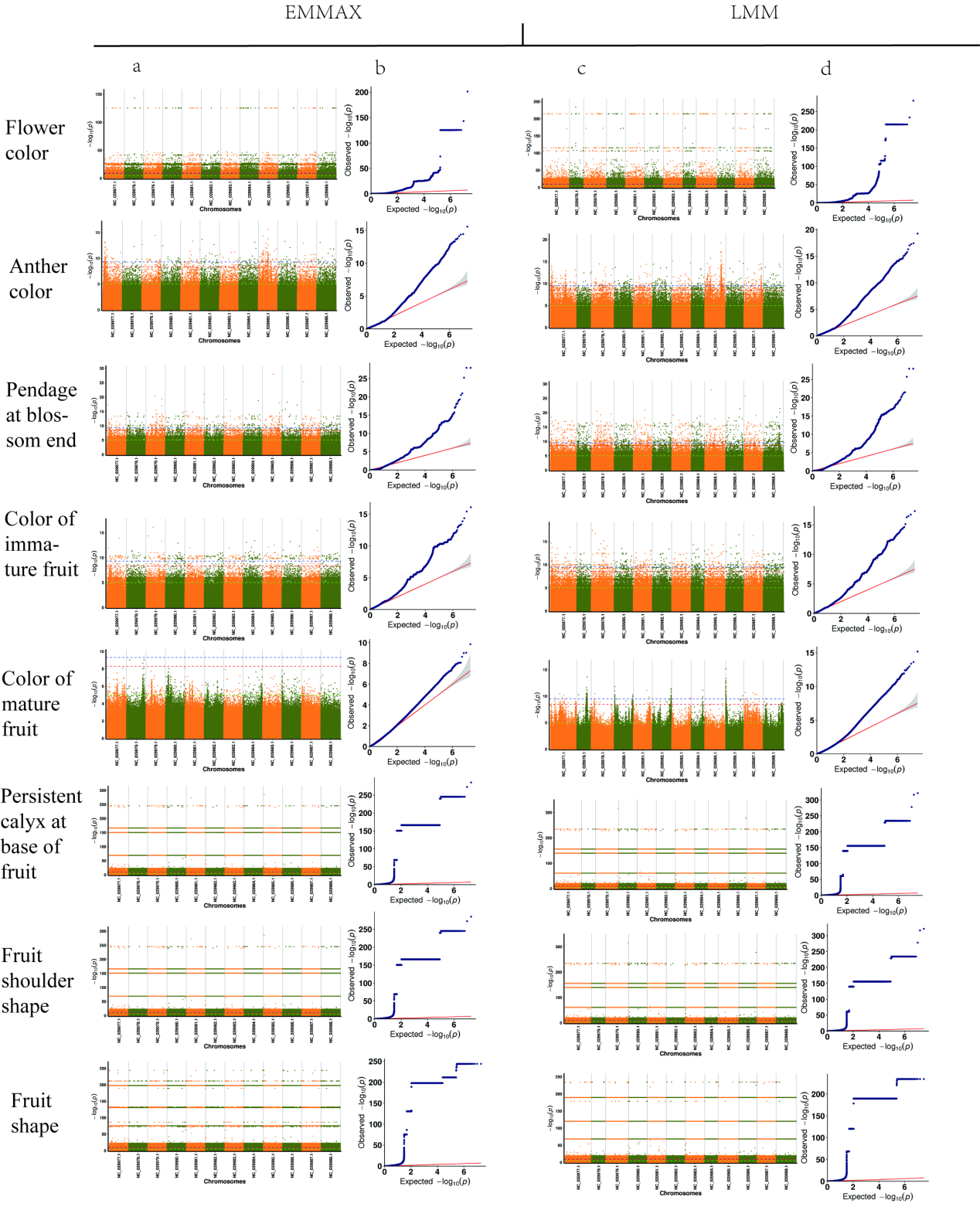


Supplementary Figure 3 Manhattan and Q-Q plots for agronomic traits related to chili peppers under the EMMAX and LMM models are presented. The horizontal axis represents chromosome position, and the vertical axis represents p-values (-log10(p)) on a base-10 logarithmic scale. Each point (or line) on the plot represents the -log10(p) value corresponding to each marker. The green horizontal line represents a value of 5 on the vertical axis, while blue or red represents values corresponding to 0.1/marker quantity and 0.01/marker quantity.

**1.2supplementary Tables**

Supplementary Table 1 Quality trait survey documentation and assignment of values

| Trait | Investigation records and assignment criteria | | | | |
| --- | --- | --- | --- | --- | --- |
|  | 0 | 1 | 2 | 3 | 4 |
| Branching type | Determinate branching | Indeterminate branching |  |  |  |
| Leaf surface | Smooth | Slightly wrinkling |  |  |  |
| Leaf shape | Lanceolate | Obvate |  |  |  |
| Style length | Shorter than stamen | Nearly the same level as stamen | Longer than stamen |  |  |
| Flower color | White | White with yellow | Light green |  |  |
| Anther color | Light purple | Yellow | Green |  |  |
| Appendage at blossom end | Absent | Present |  |  |  |
| Color of immature fruit | Milk yellow | Light green | Green | Dark green | Yellowish green |
| Color of mature fruit | Tangerine | Bright red | Dark red |  |  |
| Persistent calyx at base of fruit | Absent | Present |  |  |  |
| Fruit shoulder shape | Pointed | Blunt |  |  |  |
| Fruit shape | Long finger-shaped | Short finger-shaped | Spherical |  |  |

Supplementary Table 2 Distribution frequency and coefficient of variation of quality traits

| Trait | Frequency of classification% | | | | | Shannon-Weaver diversity index (H’) |
| --- | --- | --- | --- | --- | --- | --- |
|  | 0 | 1 | 2 | 3 | 4 |  |
| Branching type | 98.9 | 1.1 |  |  |  | 0.06 |
| Leaf surface | 41.2 | 58.8 |  |  |  | 0.68 |
| Leaf shape | 41.2 | 58.8 |  |  |  | 0.68 |
| Style length | 19.2 | 20.9 | 59.9 |  |  | 0.95 |
| Flower color | 41.8 | 0.5 | 57.7 |  |  | 0.71 |
| Anther color | 89.6 | 6.0 | 4.4 |  |  | 0.24 |
| pendage at blossom end | 96.2 | 3.8 |  |  |  | 0.16 |
| Color of immature fruit | 4.4 | 4.9 | 72.0 | 14.3 | 4.4 | 0.94 |
| Color of mature fruit | 18.1 | 75.3 | 6.6 |  |  | 0.70 |
| Persistent calyx at base of fruit | 99.5 | 0.5 |  |  |  | 0.03 |
| Fruit shoulder shape | 99.5 | 0.5 |  |  |  | 0.03 |
| Fruit shape | 40.7 | 58.8 | 0.5 |  |  | 0.71 |

Supplementary Table 3 Analysis of genetic diversity in quantitative traits

| Trait | Max | Min | Range | Mean | SD | CV(%) | Shannon-Weaver diversity index (H’) |
| --- | --- | --- | --- | --- | --- | --- | --- |
| Plant height/cm | 122.20 | 40.50 | 81.70 | 77.05 | 16.03 | 20.81 | 2.07 |
| Plant breadth/cm | 124.30 | 50.90 | 73.40 | 78.54 | 16.37 | 20.84 | 2.02 |
| Leaf length/cm | 13.57 | 3.72 | 9.85 | 8.34 | 2.01 | 24.10 | 2.07 |
| Leaf width/cm | 6.83 | 1.18 | 5.65 | 3.90 | 1.08 | 27.58 | 2.07 |
| Petiole length/cm | 10.25 | 1.22 | 9.03 | 2.83 | 1.10 | 38.83 | 1.70 |
| First stem node length/cm | 8.40 | 1.50 | 6.90 | 4.78 | 1.29 | 26.98 | 2.04 |
| Stem thickness/mm | 22.80 | 9.21 | 13.59 | 16.87 | 2.70 | 16.02 | 2.07 |
| Weight per fruit/g | 5.46 | 0.23 | 5.24 | 1.44 | 1.25 | 86.35 | 1.55 |
| Longitudinal diameter of fruit/mm | 83.87 | 15.48 | 68.38 | 37.10 | 18.86 | 50.85 | 1.82 |
| Transverse diameter of fruit/mm | 19.48 | 3.89 | 15.59 | 7.46 | 2.06 | 27.61 | 1.92 |
| Fruit peduncle length/mm | 47.21 | 21.43 | 25.78 | 31.87 | 4.68 | 14.70 | 2.03 |
| Thickness of flesh/mm | 2.61 | 0.20 | 2.41 | 0.85 | 0.43 | 50.05 | 1.88 |
| Plancenta size/mm | 3.47 | 0.19 | 3.28 | 0.90 | 0.71 | 78.76 | 1.55 |
| Number of locules | 3.00 | 2.00 | 1.00 | 2.29 | 0.44 | 19.05 | 0.95 |

Supplementary Table 4 Quantitative trait correlation analysis

| Trait | Plant height/cm | Plant breadth/cm | Leaf length/cm | Leaf width/cm | Petiole length/cm | First stem node length/cm | Stem thickness/mm | Weight per fruit/g | Longitudinal diameter of fruit/mm | Fruit peduncle length/mm | Fruit peduncle length/mm | Thickness of flesh/mm | Plancenta size/mm | Number of locules |
| --- | --- | --- | --- | --- | --- | --- | --- | --- | --- | --- | --- | --- | --- | --- |
| Plant height/cm | 1 |  |  |  |  |  |  |  |  |  |  |  |  |  |
| Plant breadth/cm | .487^**^ | 1 |  |  |  |  |  |  |  |  |  |  |  |  |
| Leaf length/cm | -.021 | -.056 | 1 |  |  |  |  |  |  |  |  |  |  |  |
| Leaf width/cm | -.081 | -.177 | .837^**^ | 1 |  |  |  |  |  |  |  |  |  |  |
| Petiole length/cm | .036 | .146^*^ | .257^**^ | .022 | 1 |  |  |  |  |  |  |  |  |  |
| First stem node length/cm | .402^**^ | .350^**^ | .065 | .013 | .025 | 1 |  |  |  |  |  |  |  |  |
| Stem thickness/mm | .122 | .054 | -.243^**^ | -.243^**^ | .107 | .036 | 1 |  |  |  |  |  |  |  |
| Weight per fruit/g | .001 | .463^**^ | -.452^**^ | -.452^**^ | .246^**^ | .082 | .374^**^ | 1 |  |  |  |  |  |  |
| Longitudinal diameter of fruit/mm | .267^**^ | .445^**^ | -.468^**^ | -.468^**^ | .246^**^ | .086 | .374^**^ | .931^**^ | 1 |  |  |  |  |  |
| Transverse diameter of fruit/mm | .185^*^ | .464^**^ | -.283^**^ | -.283^**^ | .168^*^ | .102 | .320^**^ | .818^**^ | .726^**^ | 1 |  |  |  |  |
| Fruit peduncle length/mm | .249^**^ | .169^*^ | .161^*^ | .161^*^ | .250^**^ | .247^**^ | -.044 | .179^*^ | .197^**^ | .142 | 1 |  |  |  |
| Thickness of flesh/mm | .219^**^ | .440^**^ | -.453^**^ | -.453^**^ | .264^**^ | .056 | .296^**^ | .802^**^ | .785^**^ | .789^**^ | .147^**^ | 1 |  |  |
| Plancenta size/mm | .288^**^ | .394^**^ | -.474^**^ | -.474^**^ | .191^*^ | .161^*^ | .395^**^ | .826^**^ | .797^**^ | .678^**^ | .193^**^ | -.772^**^ | 1 |  |
| Number of locules | -.037 | -.107 | .373^**^ | .373^**^ | -.098 | .097 | -.183^*^ | -.375^**^ | -.365^**^ | -.288^**^ | .151^**^ | -.473^**^ | -.343^**^ | 1 |

**. Highly significant correlation at the .01 level (bilaterally).

*. Significantly correlated at the .05 level (bilateral).

**Supplementary Table 5** Sequencing quality and comparison results statistics. of 182 pepper accessions.(shown in excel)

**Supplementary Table 6** Sample clustering information at each value of k and admixture cross-validation error rate for each value of k.(shown in excel)

**Supplementary Table 7** Significant loci and candidate gene information for 24 agronomic traits.(shown in excel)

**Supplementary Table 8** Annotation of important candidate genes for 24 agronomic traits.(shown in excel)

**Supplementary Table 9** The species and phenotypic data of 182 pepper germplasms were collected in this study.(shown in excel)
